# Supplementary material for: Preliminary Identification of Potential Vaccine Targets for the COVID-19 Coronavirus (SARS-CoV-2) Based on SARS-CoV Immunological Studies
Source: Viruses. 2020 Feb 25;12(3):254. doi: 10.3390/v12030254 (PMC7150947; doi:10.3390/v12030254)
Supplement: Supplementary file 1 [file viruses-12-00254-s001.zip › Supplementary Figure S2.pdf]

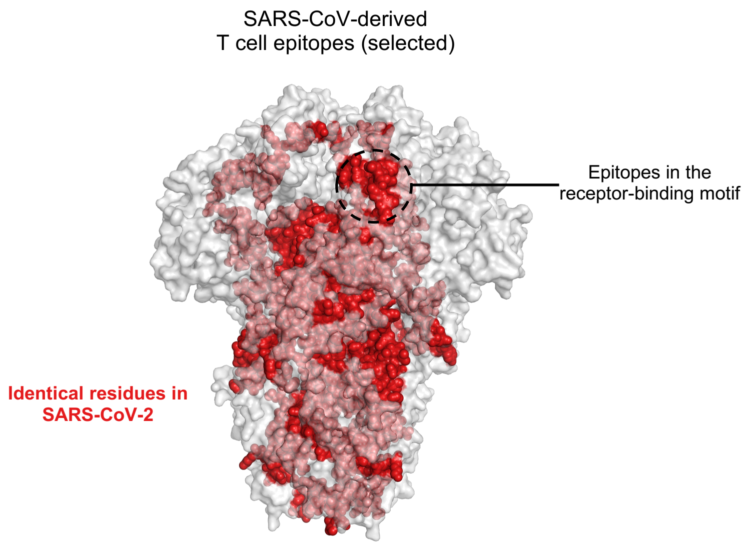

Figure S2. Location of identified T cell epitopes on the SARS-CoV S protein structure (PDB ID: 5XLR). Residues of the SARS-CoV-derived T cell epitopes (determined using positive MHC binding assays and that were identical in SARS-CoV-2) are shown in red color. The dark and light shade reflect the surface and buried residues, respectively. The 3 overlapping epitopes (GYQPYRVVVL, QPYRVVVLSF, PYRVVVLSF) that lie within the SARS-CoV receptor-binding motif are indicated by the dashed circle.
