# Supplementary material for: Preliminary Identification of Potential Vaccine Targets for the COVID-19 Coronavirus (SARS-CoV-2) Based on SARS-CoV Immunological Studies
Source: Viruses. 2020 Feb 25;12(3):254. doi: 10.3390/v12030254 (PMC7150947; doi:10.3390/v12030254)
Supplement: Supplementary file 1 [file viruses-12-00254-s001.zip › Supplementary Figure S3.pdf]

```
MFIFLLFLTLTSGSDLDRCTTFDDVQAPNYTQHTSSMRGVYYPDEIFRSDTLYLTQDLFLPFYSNVTGFH 70
MFVFLVLLPLVSSQCVNLTTRTQ--LPPAYTN--SFTRGVYYPDKVFRSSVLHSTQDLFLPFFSNVTWFH 70
** **  * * *       *       * **   *  *******  ***  *  ******** **** **

TINHT-------FGNPVIPFKDGIYFAATEKSNVVRGWVFGSTMNNKSQSVIIINNSTNVVIRACNFELC 140
AIHVSGTNGTKRFDNPVLPFNDGVYFASTEKSNIIRGWIFGTTLDSKTQSLLIVNNATNVVIKVCEFQFC 140
 *          * *** ** ** *** *****  *** ** *   * **  * ** *****  * *  *

DNPFFAV----SKPMGTQTHTMIFDNAFNCTFEYISDAFSLDVSEKSGNFKHLREFVFKNKDGFLYVYKG 210
NDPFLGVYYHKNNKSWMESEFRVYSSANNCTFEYVSQPFLMDLEGKQGNFKNLREFVFKNIDGYFKIYSK 210
  **  *                   * ****** *  *  *   * **** ******** **    *

YQPIDVVRDLPSGFNTLKPIFKLPLGINITNFRAIL----TAFSPAQDI--WGTSAAAYFVGYLKPTTFM 280
HTPINLVRDLPQGFSALEPLVDLPIGINITRFQTLLALHRSYLTPGDSSSGWTAGAAAYYVGYLQPRTFL 280
  **  ***** **  * *   ** ***** *   *        *      *   **** **** * **

LKYDENGTITDAVDCSQNPLAELKCSVKSFEIDKGIYQTSNFRVVPSGDVVRFPNITNLCPFGEVFNATK 350
LKYNENGTITDAVDCALDPLSETKCTLKSFTVEKGIYQTSNFRVQPTESIVRFPNITNLCPFGEVFNATR 350
*** ***********   ** * **  ***   *********** *    *******************

FPSVYAWERKKISNCVADYSVLYNSTFFSTFKCYGVSATKLNDLCFSNVYADSFVVKGDDVRQIAPGQTG 420
FASVYAWNRKRISNCVADYSVLYNSASFSTFKCYGVSPTKLNDLCFTNVYADSFVIRGDEVRQIAPGQTG 420
* ***** ** **************  ********** ******** ********  ** **********

VIADYNYKLPDDFMGCVLAWNTRNIDATSTGNYNYKYRYLRHGKLRPFERDISNVPFSPDGKPCTP-PAL 490
KIADYNYKLPDDFTGCVIAWNSNNLDSKVGGNYNYLYRLFRKSNLKPFERDISTEIYQAGSTPCNGVEGF 490
 ************ *** ***  * *    ***** **  *   * *******         **

NCYWPLNDYGFYTTTGIGYQPYRVVVLSFELLNAPATVCGPKLSTDLIKNQCVNFNFNGLTGTGVLTPSS 560
NCYFPLQSYGFQPTNGVGYQPYRVVVLSFELLHAPATVCGPKKSTNLVKNKCVNFNFNGLTGTGVLTESN 560
*** **  ***  * * *************** ********* ** * ** **************** *

KRFQPFQQFGRDVSDFTDSVRDPKTSEILDISPCAFGGVSVITPGTNASSEVAVLYQDVNCTDVSTAIHA 630
KKFLPFQQFGRDIADTTDAVRDPQTLEILDITPCSFGGVSVITPGTNTSNQVAVLYQDVNCTEVPVAIHA 630
* * ********  * ** **** * ***** ** ************ *  *********** *  ****

DQLTPAWRIYSTGNNVFQTQAGCLIGAEHVDTSYECDIPIGAGICASYHTVS----LLRSTSQKSIVAYT 700
DQLTPTWRVYSTGSNVFQTRAGCLIGAEHVNNSYECDIPIGAGICASYQTQTNSPRRARSVASQSIIAYT 700
***** ** **** ***** **********  **************** *        **    ** ***

MSLGADSSIAYSNNTIAIPTNFSISITTEVMPVSMAKTSVDCNMYICGDSTECANLLLQYGSFCTQLNRA 770
MSLGAENSVAYSNNSIAIPTNFTISVTTEILPVSMTKTSVDCTMYICGDSTECSNLLLQYGSFCTQLNRA 770
*****  * ***** ******* ** ***  **** ****** ********** ****************

LSGIAAEQDRNTREVFAQVKQMYKTPTLKYFGGFNFSQILPDPLKPTKRSFIEDLLFNKVTLADAGFMKQ 840
LTGIAVEQDKNTQEVFAQVKQIYKTPPIKDFGGFNFSQILPDPSKPSKRSFIEDLLFNKVTLADAGFIKQ 840
* *** *** ** ******** ****  * ************* ** ******************** **

YGECLGDINARDLICAQKFNGLTVLPPLLTDDMIAAYTAALVSGTATAGWTFGAGAALQIPFAMQMAYRF 910
YGDCLGDIAARDLICAQKFNGLTVLPPLLTDEMIAQYTSALLAGTITSGWTFGAGAALQIPFAMQMAYRF 910
** ***** ********************** *** ** **  ** * **********************

NGIGVTQNVLYENQKQIANQFNKAISQIQESLTTTSTALGKLQDVVNQNAQALNTLVKQLSSNFGAISSV 980
NGIGVTQNVLYENQKLIANQFNSAIGKIQDSLSSTASALGKLQDVVNQNAQALNTLVKQLSSNFGAISSV 980
*************** ****** **  ** **  *  *********************************

LNDILSRLDKVEAEVQIDRLITGRLQSLQTYVTQQLIRAAEIRASANLAATKMSECVLGQSKRVDFCGKG 1050
LNDILSRLDKVEAEVQIDRLITGRLQSLQTYVTQQLIRAAEIRASANLAATKMSECVLGQSKRVDFCGKG 1050
**********************************************************************

YHLMSFPQAAPHGVVFLHVTYVPSQERNFTTAPAICHEGKAYFPREGVFVFNGTSWFITQRNFFSPQIIT 1120
YHLMSFPQSAPHGVVFLHVTYVPAQEKNFTTAPAICHDGKAHFPREGVFVSNGTHWFVTQRNFYEPQIIT 1120
******** ************** ** ********** *** ******** *** ** *****  *****

TDNTFVSGNCDVVIGIINNTVYDPLQPELDSFKEELDKYFKNHTSPDVDLGDISGINASVVNIQKEIDRL 1190
TDNTFVSGNCDVVIGIVNNTVYDPLQPELDSFKEELDKYFKNHTSPDVDLGDISGINASVVNIQKEIDRL 1190
**************** *****************************************************

NEVAKNLNESLIDLQELGKYEQYIKWPWYVWLGFIAGLIAIVMVTILLCCMTSCCSCLKGACSCGSCCKF 1260
NEVAKNLNESLIDLQELGKYEQYIKWPWYIWLGFIAGLIAIVMVTIMLCCMTSCCSCLKGCCSCGSCCKF 1260
***************************** **************** ************* *********

DEDDSEPVLKGVKLHYT 1277
DEDDSEPVLKGVKLHYT 1277
*****************
```

Figure S3. Pairwise sequence alignment of the reference sequences of the S proteins of SARS-CoV and SARS-CoV-2 (accession ID: NP_828851.1 and YP_009724390.1, respectively). Identical residues are indicated by *.
