# Supplementary material for: Preliminary Identification of Potential Vaccine Targets for the COVID-19 Coronavirus (SARS-CoV-2) Based on SARS-CoV Immunological Studies
Source: Viruses. 2020 Feb 25;12(3):254. doi: 10.3390/v12030254 (PMC7150947; doi:10.3390/v12030254)
Supplement: Supplementary file 1 [file viruses-12-00254-s001.zip › Supplementary Figure S1.pdf]

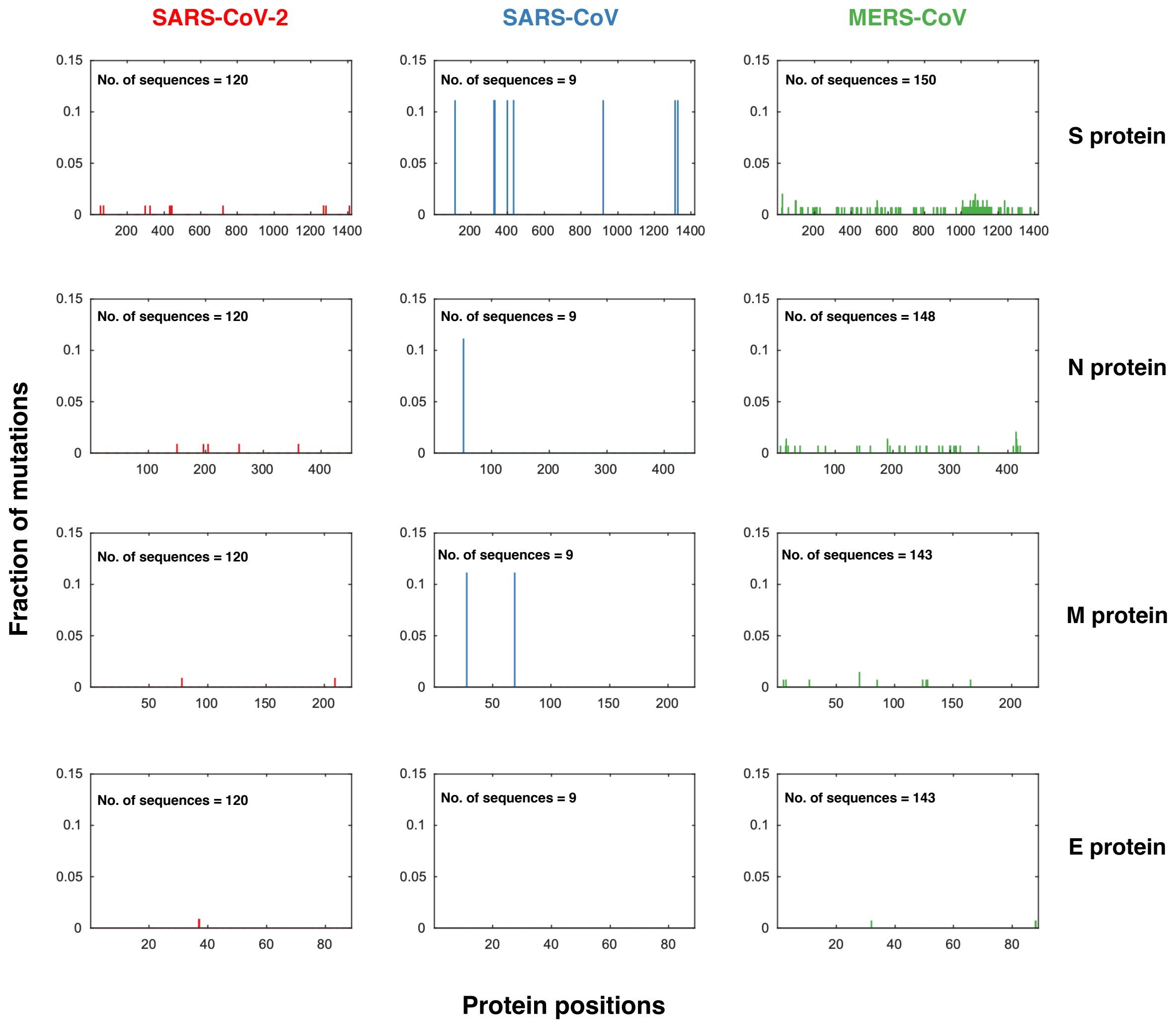

Figure S1. Fraction of mutations in the observed sequences of the structural proteins of the three coronaviruses. Mutation is defined here as an amino acid difference from the reference sequence of the respective coronavirus; accession IDs: NC_045512.2 (2019-nCoV), NC_004718.3 (SARS-CoV), and NC_019843.3 (MERS-CoV).
